# Halofuginone and artemisinin synergistically arrest cancer cells at the G1/G0 phase by upregulating p21^Cip1^ and p27^Kip1^

**DOI:** 10.18632/oncotarget.10367

**Published:** 2016-07-01

**Authors:** Guoqing Chen, Ruihong Gong, Xianli Shi, Dajian Yang, Ge Zhang, Aiping Lu, Jianbo Yue, Zhaoxiang Bian

**Affiliations:** ^1^ School of Chinese Medicine, Hong Kong Baptist University, Hong Kong, China; ^2^ Chongqing Academy of Chinese Materia Medica, Chongqing, China; ^3^ Department of Biomedical Sciences, City University of Hong Kong, Hong Kong, China

**Keywords:** halofuginone, artemisinin, synergy, cell proliferation, cell cycle

## Abstract

Combinational drug therapy is one of the most promising strategies in modern anticancer research. Traditional Chinese medicine (TCM) formulas represent a wealth of complex combinations proven successful over centuries of clinical application. One such formula used to treat a variety of diseases, including cancer, contains two herbs, whose main active components are Halofuginone (HF) and Artemisinin (ATS). Here we studied the anticancer synergism of HF and ATS in various cancer cell lines and in a xenograft nude mice model. We found that the HF-ATS combination arrested more cells at the G1/G0 phase than either one alone, with the concomitant increased levels of CDK2 inhibitors, p21^Cip1^ and p27^Kip1^. By knocking down p21^Cip1^ and p27^Kip1^ separately or simultaneously in HCT116 cells and MCF-7 cells, we found that p21^Cip1^ was required for HF induced G1/G0 arrest, whereas p21^Cip1^ and p27^Kip1^ were both required for ATS or HF-ATS combination-mediated cell cycle arrest. Moreover, HF-ATS combination synergistically inhibited tumor growth in xenograft nude mice, and this was associated with the increased levels of p21^Cip1^ and p27^Kip1^. Collectively, these data indicate that the upregulation of p21^Cip1^ and p27^Kip1^ contributes to the synergistic anticancer effect of the HF-ATS combination.

## INTRODUCTION

Cancer is a complex disease; it affects various tissues in multiple ways and has many subtypes [1]. Although there are various anticancer drugs, they often have high toxicity, low efficacy, and could lead to drug resistance after prolonged usage. One possible reason for this phenomenon is that majority of the current anticancer drugs were designed to target a single pathway or protein, while cancer is often the result of an incredibly complex combination of deregulated signaling pathways [2]. Drug developers have observed that combinations of some anticancer drugs exhibit synergistic effects, presumably via simultaneously targeting multiple pathways. This multi-pronged effect could lead to maximal therapeutic efficacy with minimal adverse effects [3, 4].

Combination therapy has been the cornerstone of herbal therapy in TCM for more than 2,500 years. Herbs are typically prescribed in formulas, based on the unique theory of TCM. In general, a formula is composed of several herbs, each of which contains several active ingredients targeting different aspect of the patient's condition. Researchers have found that the combination of the main active components from the herbs in a formula can be as effective as the formula, or even better [5, 6]. ATS and HF are two active compounds coming from *Artemisia carvifolia* (Qinghao in Chinese) and *Dichroa febrifuga Lour* (Changshan in Chinese), respectively, which are two herbs commonly used together as a formula to treat a variety of diseases in Chinese folk medicine, including cancer. In recent years, both ATS and HF have been intensively studied because of their potential therapeutic effects in cancer treatment. For example, ATS has anti-proliferation effects on human breast cancer [7], neuroblastoma [8] and ishikawa endometrial cancer [9]. HF also has the ability to inhibit the proliferation of human colorectal cancer cells [10], multiple myeloma cells [11], and liver cancer cells [12]. According to the history of using the formula of Qinghao and Changshan in TCM, we hypothesize that HF and ATS could exhibit synergistic anticancer effect. However, to the best of our knowledge, there is no published research on the synergistic effect of HF and ATS on inhibiting cancer cells growth.

Here we applied the Chou-Talalay Method of analysis [13] and found that HF-ATS combination exhibited synergistic anticancer effects in a variety of human cancer cell lines, and in a xenograft nude mice model. Furthermore, we found that the combination of HF and ATS arrested various human cancer cells at G1/G0 phase, suggesting that the cross-talk in key signaling pathways or key proteins may exist between these two compounds. The cell cycle in cancer cells is often deregulated resulting in uncontrolled cell proliferation [14, 15], thus inhibiting the cell cycle is a viable strategy for treating cancer [16, 17]. Therefore, we speculate that the HF-ATS combination synergistically arrests cancer cells at G1/G0 phase by cooperatively regulating one or two key cell cycle regulatory proteins. In this study, we constructed p21^Cip1^, or p27^Kip1^, or p21^Cip1^-p27^Kip1^ double knockdown cancer cell lines. Using these knockdown cancer cell lines and the animal model, we demonstrated that the HF-ATS combination exhibits the synergistic anticancer activity by upregulating p21^Cip1^ and p27^Kip1^ cooperatively to arrest cells at G1/G0 phase both *in vivo* and *in vitro*.

## RESULTS

### The HF-ATS combination synergistically inhibits the proliferation of cancer cells

**Figure 1:**
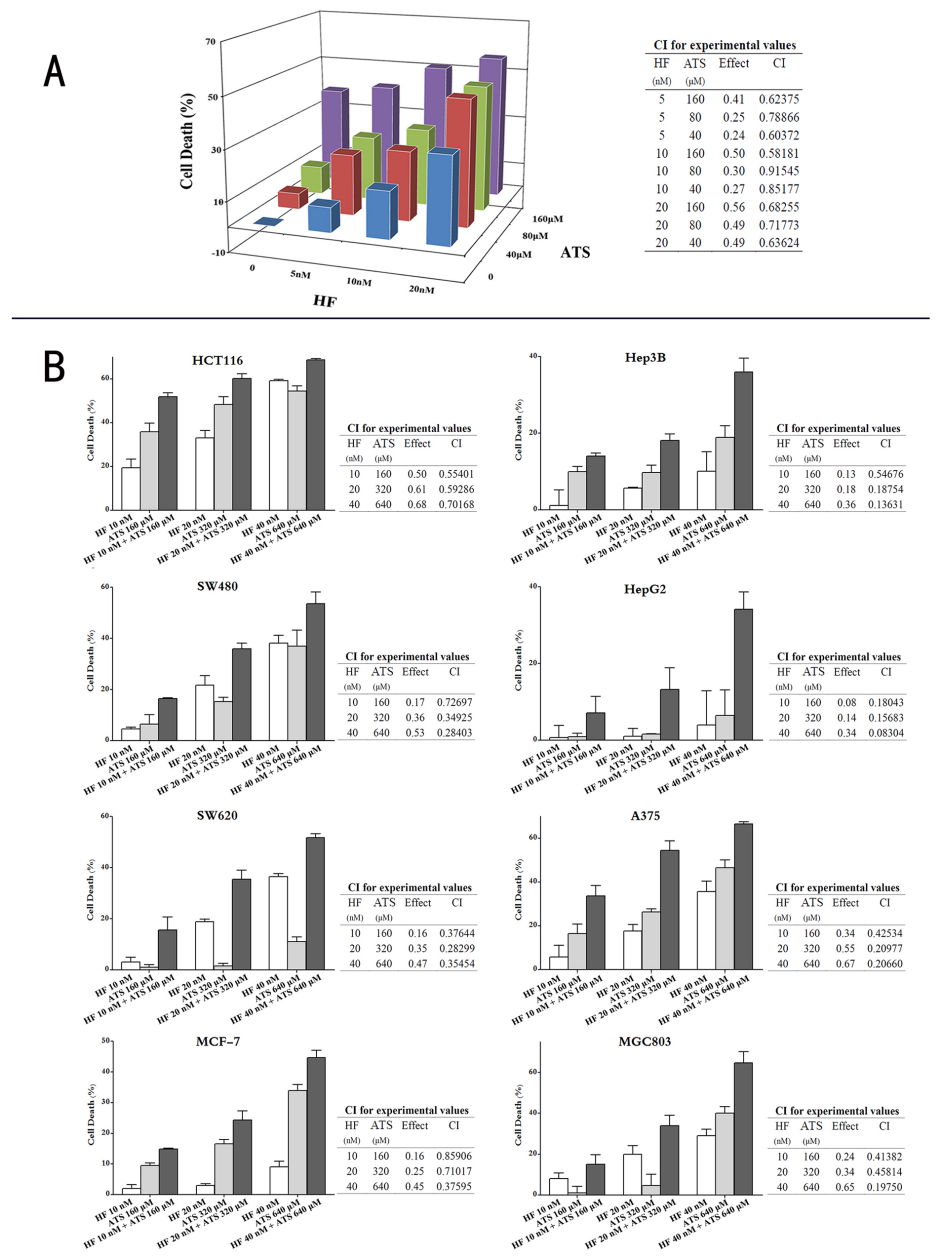
HF-ATS combination synergistically inhibited the proliferation of cancer cells **A.** HCT116 cells were treated with different dose of HF (5, 10, 20 nM), ATS (40, 80, 160 μM), or combinations of both. 24 h later, cell viabilities were measured by MTT assay. Combination indexes (CIs) were analyzed by the CompuSyn software. CI< 1 indicates synergism. **B.** Eight cancer cell lines were treated with increasing concentrations of HF-ATS combinations with the selected optimal fixed ratio. 24 h later, cell viabilities were measured by MTT assay. Combination indexes (CIs) were analyzed by using the CompuSyn software. CI < 1 indicates synergism.

To test the synergistic anticancer activity of HF and ATS in different cancer cell lines, firstly we performed a MTT assay to screen the best combination ratio with three-dimensional analysis [18]. HCT116 cells were cultured in a 96-well plate and treated with increasing concentrations of HF (5 nM to 20 nM) and ATS (40 μM to 160 μM) alone or together. We then applied the CompuSyn program, which is based on the theory of Chou-Talalay Method, to assess the effects of the diverse combinations. The combination index (CI) < 1, = 1, and > 1, represents synergistic, additive, and antagonistic effects, respectively [19]. As shown in Figure 1A, all the CIs of different ratios in HCT116 cells were < 1, indicating a synergism between HF and ATS. Among these combinations, the 10 nM HF combined with 160 μM ATS exhibited the lowest CI but the second highest anti-proliferation potency, indicating that this ratio of HF-ATS combination is the best. Next, a series of dose-dependent HF-ATS combinations with the fixed ratio were tested in eight cancer cell lines. We found these combinations of the two compounds significantly decreased cell viability in different cancer cell lines compared with the single agents alone, and their CIs in different cancer cells were all < 1 (Figure 1B). Among them, HCT116 was the most sensitive cell line in response to the HF-ATS combination. In summary, these results indicate that HF and ATS indeed exhibit a synergistic anticancer effect.

### HF–ATS combination arrests cell cycle at the G1/G0 phase

**Figure 2:**
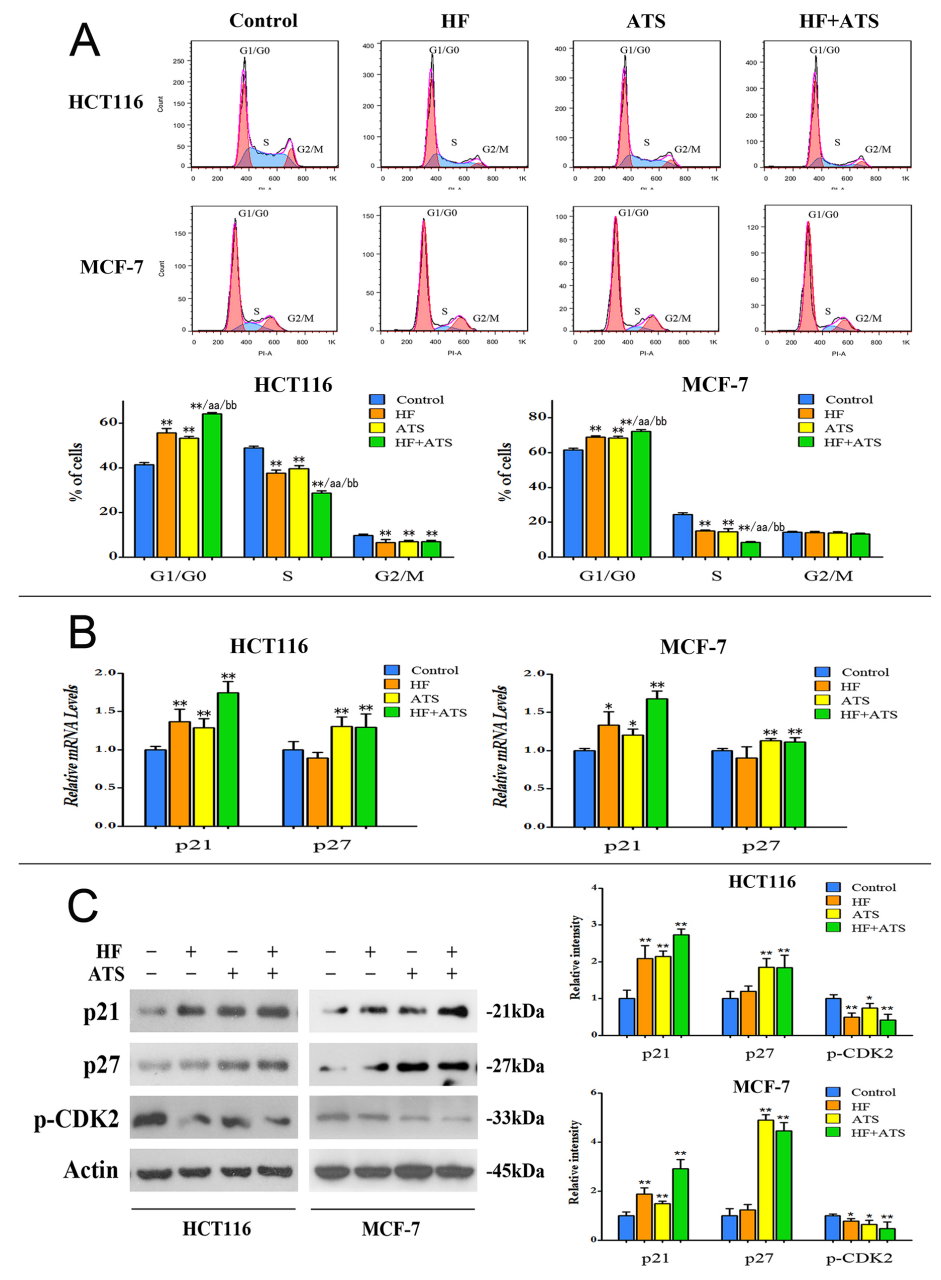
HF–ATS combination markedly arrested cells at the G1/G0 phase **A.** Flow cytometry analysis of HCT116 cells or MCF-7 cells treated with combination of HF (10 nM) and ATS (160 μM) for 12 h or 24 h, respectively, which cooperatively induced cell cycle arrest at the G1/G0 phase. *P < 0.05, **P < 0.01, compared with control group; aa P < 0.01, compared with HF; bb P < 0.01, compared with ATS. **B.** Quantitative RT-PCR analysis of p21^Cip1^ and p27^Kip1^ mRNA in HCT116 cells or MCF-7 cells treated with the combination of HF (10 nM) and ATS (160 μM) for 12 h or 24 h, respectively. *P < 0.05, **P < 0.01, compared with control group. **C.** Protein expression of p21^Cip1^, p27^Kip1^ and phospho-CDK2 in HCT116 cells or MCF-7 cells (left panel); quantitative analysis of protein expressions (right panel) treated with the combination of HF (10 nM) and ATS (160 μM) for 12 h or 24 h, respectively.

Since HF–ATS combination showed stronger growth-inhibitory effect on various cancer cells compared with single agents, we next investigated whether HF and ATS alone or together had similar inhibitory effects on cell cycle progression. We found that treatment of HCT116 cells or MCF-7 cells with HF or ATS arrested more cells at G1/G0 phase as compared with the untreated control cells, and an even higher percentage of cells were arrested at the G1/G0 phase after treatment of HF-ATS combination (Figure 2A).

In order to confirm those results, HCT116 cells or MCF-7 cells were synchronized by serum starvation-release experiments. Generally, HCT116 cells or MCF-7 cells can be synchronized at the G1/G0 phase by serum starvation for 24 h or 48 h, respectively. After serum starvation, these cells re-enter the cell cycle after the addition of serum. HCT116 cells or MCF-7 cells were successfully arrested at the G1/G0 phase by starvation as shown in Figure S1. After serum addition, HCT116 cells or MCF-7 cells re-entered the cell cycle, with gradual decrease of G1 phase and increase of S phase fraction. However, treatment of cells with HF or ATS markedly inhibited the progression of cells from G1/G0 phase to S phase transition. This defect was significantly more severe after treatment with HF-ATS combination (Figure S1). These results support our hypothesis that HF-ATS combination cooperatively arrests cells at the G1/G0 phase, thereby inhibiting the proliferation of cancer cells.

### HF up-regulates only p21^Cip1^, while ATS up-regulates p21^Cip1^ and p27^Kip1^ in cancer cells

Because CDK2 and CDK2 inhibitors, such as p21^Cip1^ and p27^Kip1^, play essential roles in regulating cell cycle progression from G1/G0 to S phase [20], we examined the effects of HF and ATS on these targets. By using RT-PCR, western blot, and immunostaining analyses, we found that treatment of cells with either HF or ATS markedly inhibited the active phosphorylation of CDK2, and the HF-ATS combination induced a more significant inhibition of CDK2 phosphorylation (Figure 2C and Figure S2). HF treatment also led to an increased level of p21^Cip1^ protein while ATS treatment alone or together with HF significantly increased the levels of both p21^Cip1^ and p27^Kip1^. Notably, p21^Cip1^ was increased more significantly in the combination treatment compared with either HF or ATS treatment (Figure 2B, 2C and Figure S2). These observations suggest that p21^Cip1^ and p27^Kip1^ are the central players in the G1/G0 phase cell cycle arrest induced by the HF-ATS combination.

### p21^Cip1^ and p27^Kip1^ are required for HF-ATS combination-induced G1/G0 arrest

**Figure 3:**
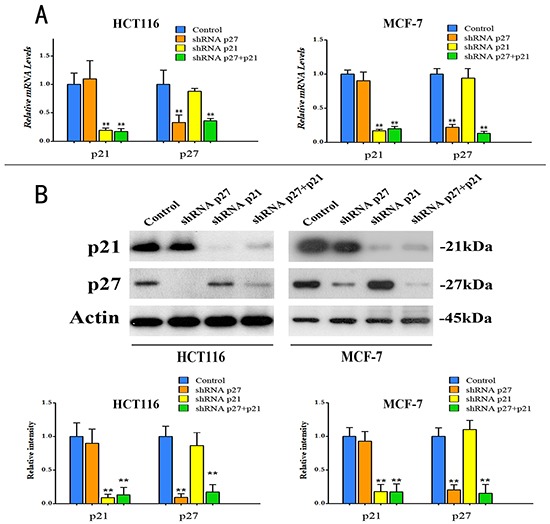
Knockdown of p21^Cip1^, or p27^Kip1^, or both in HCT116 cells and MCF-7 cells **A.** Quantitative RT-PCR analysis of p21^Cip1^ mRNA and p27^Kip1^ mRNA in the knockdown HCT116 cells and MCF-7 cells. **P < 0.01, compared with control group. **B.** Protein expression levels of p21^Cip1^ and p27^Kip1^ (upper panel), and quantitative analysis of protein expressions (bottom panel) in knockdown HCT116 cells and MCF-7 cells.

To dissect the roles of p21^Cip1^ and p27^Kip1^ in the synergistic effect of HF and ATS on cell cycle arrest, we constructed the p21^Cip1^ knockdown, p27^Kip1^ knockdown, and p21^Cip1^-p27^Kip1^ double knockdown HCT116 cells or MCF-7 cells. The knockdown efficiencies were confirmed by RT-PCR and western blot analyses (Figure 3A and 3B).

**Figure 4:**
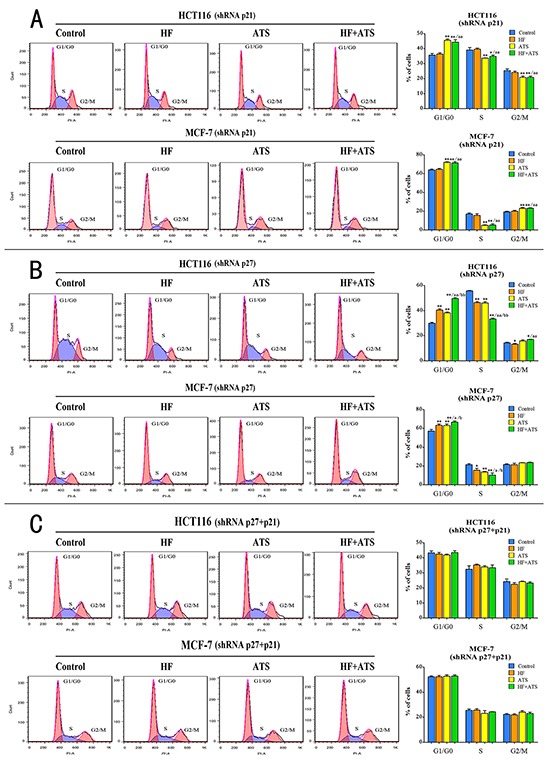
Both p21^Cip1^ and p27^Kip1^ are required for HF-ATS combination-induced cell cycle arrest **A.** Flow cytometry (left panel) and histogram (right panel) analyses of p21^Cip1^ knockdown HCT116 cells or p21^Cip1^ knockdown MCF-7 cells treated with the combination of HF (10 nM) and ATS (160 μM) for 12 h or 24 h, respectively. **B.** Flow cytometry (left panel) and histogram (right panel) analyses of p27^Kip1^ knockdown HCT116 cells or p27^Kip1^ knockdown MCF-7 cells treated with the combination of HF (10 nM) and ATS (160 μM) for 12 h or 24 h, respectively. **C.** Flow cytometry (left panel) and histogram (right panel) analyses of p21^Cip1^-p27^Kip1^ double knockdown HCT116 cells or p21^Cip1^-p27^Kip1^ double knockdown MCF-7 cells treated with the combination of HF (10 nM) and ATS (160 μM) for 12 h or 24 h, respectively. *P < 0.05, **P < 0.01, compared with control group; a P < 0.05, aa P < 0.01, compared with HF; b P < 0.05, bb P < 0.01, compared with ATS.

Subsequently, we analyzed the effects of HF-ATS combination on the G1/G0 to S phase transition in these knockdown cell lines. In p21^Cip1^ knockdown HCT116 cells or MCF-7 cells, ATS alone or together with HF still induced the G1/G0 cell cycle arrest but HF alone did not (Figure 4A). Similar results were observed in the serum starvation-release experiment (Figure S3A). In p27^Kip1^ knockdown HCT116 cells or MCF-7 cells, ATS and HF alone or together also induced cell cycle arrest at the G1/G0 phase. Moreover, more cells were arrested at the G1/G0 phase in both cancer cells treated with HF–ATS combination compared with single agent treatment (Figure 4B). Similar results were also observed in the serum starvation-release experiment (Figure S3B). However, in p21^Cip1^-p27^Kip1^ double knockdown cancer cells, neither HF nor ATS induced G1/G0 cell cycle arrest. Even after HF-ATS combination treatment, there was no significant difference in cell cycle progression as compared with the control (Figure 4C). Similar results were observed in the serum starvation synchronization experiments in p21^Cip1^-p27^Kip1^ double knockdown cells (Figure S3C).

**Figure 5:**
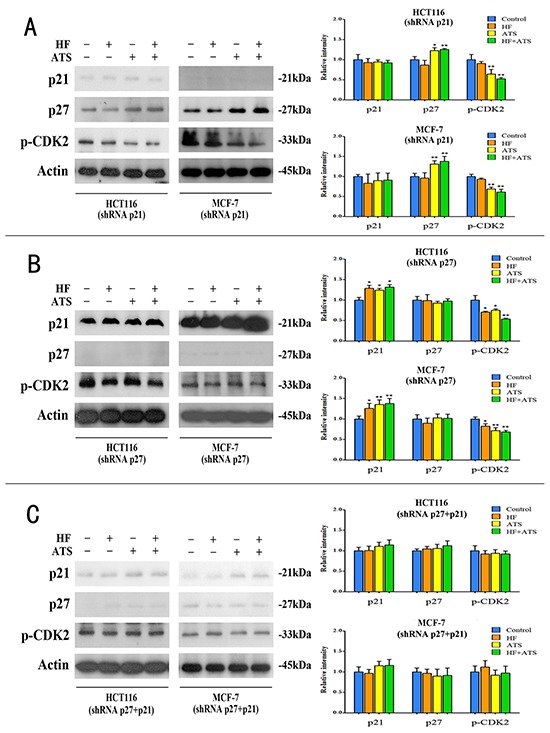
HF, ATS and HF-ATS combination differentially modulated p21^Cip1^, p27^Kip1^ and phospho-CDK2 in p21^Cip1^ or/and p27^Kip1^ knockdown cells **A.** The expression of p21^Cip1^, p27^Kip1^, and phospho-CDK2 by combination of HF (10 nM) and ATS (160 μM) (left panel), and quantitative analysis of protein expressions (right panel) in p21^Cip1^ knockdown HCT116 cells or p21^Cip1^ knockdown MCF-7 cells. **B.** The expression of p21^Cip1^, p27^Kip1^ and phospho-CDK2 by combination of HF (10 nM) and ATS (160 μM) (left panel), and quantitative analysis of protein expressions (right panel) in p27^Kip1^ knockdown HCT116 cells or p27^Kip1^ knockdown MCF-7 cells. **C.** Expression of p21^Cip1^, p27^Kip1^ and phospho-CDK2 by combination of HF (10 nM) and ATS (160 μM) (left panel), and quantitative analysis of protein expressions (right panel) in p21^Cip1^-p27^Kip1^ double knockdown HCT116 cells or p21^Cip1^-p27^Kip1^ double knockdown MCF-7 cells.

To further confirm whether p21^Cip1^ and p27^Kip1^ are the central players in the G1/G0 arrest induced by HF–ATS combination, we performed RT-PCR, western blot and immunostaining analyses on these knockdown cell lines. In p21^Cip1^ knockdown HCT116 cells or MCF-7 cells, ATS still up-regulated the expression of p27^Kip1^ and inhibited the active phosphorylation of CDK2. HF-ATS combination treatment gave the similar result (Figure 5A, Figure S4A and S5). In p27^Kip1^ knockdown HCT116 cells or MCF-7 cells, HF or ATS also up-regulated the expression of p21^Cip1^ and inhibited the active phosphorylation of CDK2, and those abnormalities were further manifested by the HF-AST combination treatment (Figure 5B, Figure S4B and S6). In contrast, in p21^Cip1^-p27^Kip1^ double knockdown cancer cells, HF, or ATS, or HF-ATS combination had no effects on the expression of the aforementioned targets (Figure 5C, Figure S4C and S7). Collectively, these results indicate that both p21^Cip1^ and p27^Kip1^ are required for the synergistic inhibitory effects of HF-ATS combination on tumor cells growth.

### HF-ATS combination synergistically inhibits tumor growth in xenograft nude mice

**Figure 6:**
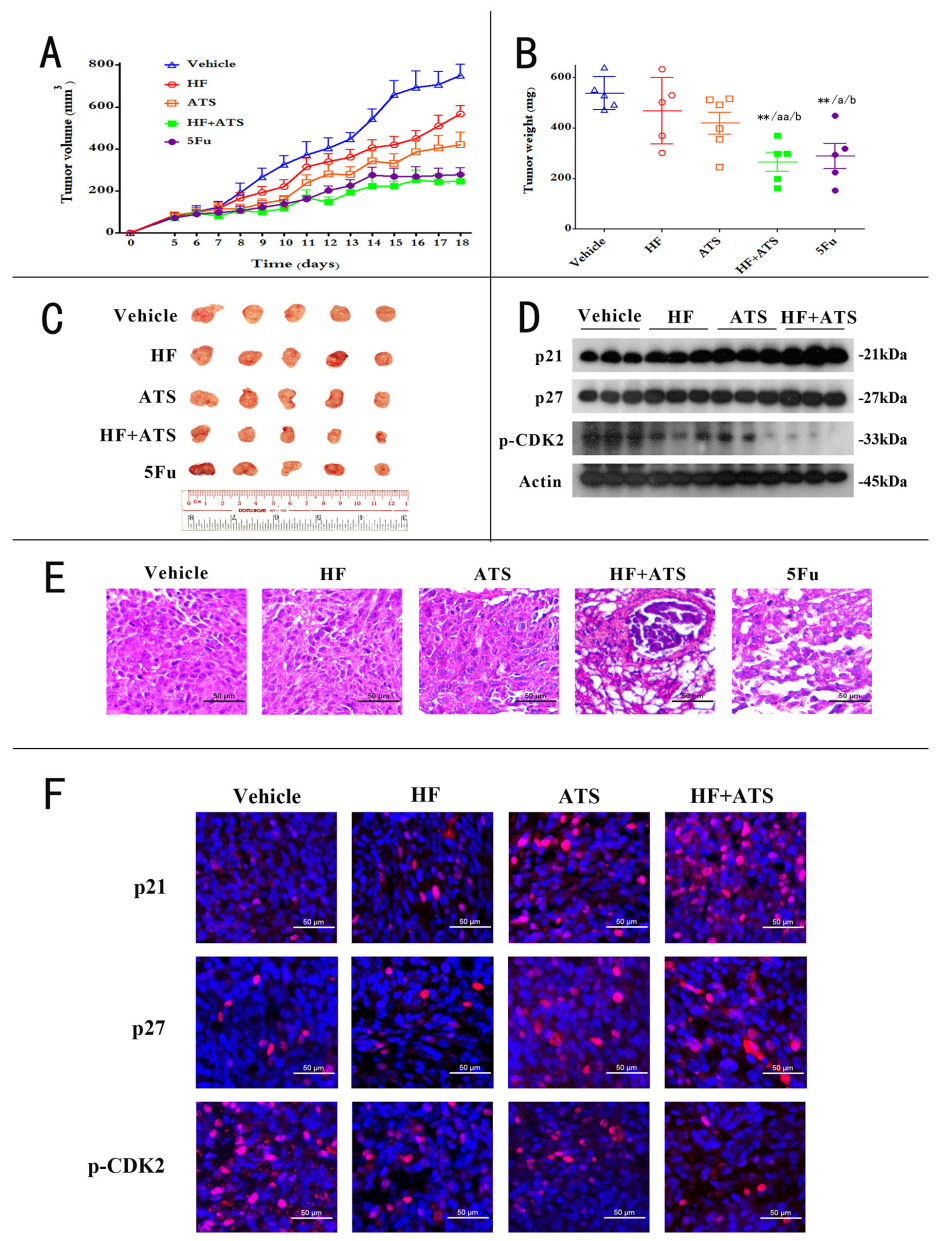
HF–ATS combination exhibited synergistic anticancer activity in CRC xenograft nude mice **A.** 6-week old nude mice were engrafted with HCT116 cells and randomly divided into 5 groups: vehicle group, HF group, ATS group, HF+ATS group, 5Fu group (n=5). Tumor volumes were measured and calculated by the length and width every day. **B.** The tumor weights of the 5 groups. *P < 0.05, **P < 0.01, compared with control group; a P < 0.05, aa P < 0.01, compared with HF; b P < 0.05, bb P < 0.01, compared with ATS. **C.** The xenograft tumors were dissected and measured. **D.** Expression levels of p21^Cip1^, p27^Kip1^ and phospho-CDK2 in xenograft tumors. **E.** H&E staining showed the decreased cell density and disrupted cell morphology in the tumor sections from combination of HF and ATS treated mice. Scale bar = 50 μm. **F.** Immunofluorescence staining for p21^Cip1^, p27^Kip1^ and phospho-CDK2 in xenograft tumors. Scale bar = 50 μm.

To determine the synergistic anticancer activity of HF-ATS combination *in vivo*, nude mice were injected with human colorectal cancer cells and then co-administrated with HF, ATS, or both. Tumor volumes and masses were increased dramatically in the control group; however, in animals exposed to the HF-ATS combination, the tumor volumes and masses were significantly smaller than those in mice treated with HF or ATS alone (Figure 6A, 6B and 6C). The anticancer ability of the HF-ATS combination was very similar to that of 5Fu, a widely used anticancer chemotherapy drug, but the bodyweight of HF-ATS combination treatment group was unaffected as compared with 5Fu, which induced bodyweight loss with the extended treatment (Figure S8). H&E staining showed that cells were densely packed in the tumor tissue of the control group, while there were many vacuoles in the tumor tissue from the HF-ATS combination treatment group. Fewer vacuoles were found in groups treated with HF or ATS alone. Similar to HF-ATS combination group, the vacuoles were also observed in the 5Fu treated group (Figure 6E). In addition, western blot and immunofluorescence staining analyses were performed to detect the expression levels of p21^Cip1^, p27^Kip1^ and phospho-CDK2 in the tumors from different groups. Although the levels of p21^Cip1^ and p27^Kip1^ were increased and the phosphorylation of CDK2 was inhibited in the AST or HF-ATS combination groups, their levels were changed more significantly in the latter group. In the HF group, only p21^Cip1^ was increased (Figure 6D and 6F). In summary, these results indicate that the synergistic inhibition of tumor growth by the HF-ATS combination is associated with the increased levels of p21^Cip1^ and p27^Kip1^ and the decreased phosphorylation of CDK2.

## DISCUSSION

In the past few years, accumulating evidence indicate that combination therapy is more effective with fewer side effects than single drug in the cancer treatment [21]. Consequently, in the pharmacological industry, there is a shift from developing drugs specifically targeting single signaling pathway or protein to combination therapies that consist of more than one active ingredients targeting multiple signaling pathways or proteins [22]. In TCM, combination therapies are the rule rather than the exception; over centuries of practice, many multi-herb formulas have been developed to effectively treat a wide spectrum of diseases and medical conditions. Given its long history of successful clinical application, TCM is a likely source of therapeutic agents for modern pharmaceutical development for many diseases, including cancer [23]. Already there has been some success of this strategy, for example, researchers have identified multiple active components from a traditional formula, and the combination of which are effective in treating acute promyelocytic leukemia (APL) [5].

In this study, we hypothesize that the combination of two ingredients, HF and ATS, derived from two herbs in a TCM formula, has a synergistic anticancer effect. In order to confirm our hypothesis, firstly we identified the best ratio of HF–ATS combination by screening with three-dimensional analysis. Subsequently, we confirmed the activities of a series of dose-dependent HF-ATS combinations with the fixed ratio in various cancer cell lines. The results showed all the CIs in different cancer cells were < 1. The CI is the natural law–based general expression of pharmacologic drug interactions, and is the simplest possible way for quantifying synergism or antagonism [13]. Our results indicated that HF and ATS indeed exhibited synergistic anticancer effect *in vitro*, and HF-ATS combination showed the highest effect in HCT116 cells. In addition, the synergistic anticancer activity of HF-ATS combination on tumor growth was validated in xenograft-bearing nude mice. Notably, in the animal model, the administrated dose of HF or ATS was much lower compared with the previous reports [10, 24, 25], while the weight and size of tumor in HF-ATS combination group were markedly smaller than either HF or ATS group alone. This result confirms that there is a synergistic anticancer effect *in vivo* for the HF-ATS combination.

It's clear that, in cancer cells, the cell cycle is often deregulated as a result of genetic mutations, which lead to uncontrolled cell proliferation [14, 15]. Therefore, inhibiting the cell cycle process is a good strategy for treating cancer, as well as other proliferative diseases [16, 17]. In this study, we found that treatment of cells with either HF or ATS arrested cancer cells at the G1/G0 phase, which was consistent with previous reports [26, 27]. Interestingly, treatment of cells with HF-ATS combination arrested more cells at the G1/G0 phase compared with either agent alone. This suggests that arrest of cells at the G1/G0 phase is the biochemical basis for the synergistic anticancer effect of the HF-ATS combination.

Cyclin-dependent kinases (CDKs) are the central components which govern the initiation, progression and completion of cell division [28]. In particular, the transition of cell cycle from the G1/G0 to the S phase is regulated by CDK2, and its excess activity is correlated with the deregulated cell proliferation rates in cancers [29]. Hence, CDK2 inhibitors are potentially effective anticancer agents [30]. p21^Cip1^ and p27^Kip1^ are two main CDK-inhibitors (CKIs); they regulate CDK2 activity by binding to cyclin-CDK complexes thereby inhibiting their catalytic activity [31]. Thus, the regulation of CDK2 through p27^Kip1^ and p21^Cip1^ plays a key role in controlling the tempo of gene transcription in G1 phase and in the subsequent progression to the cell division [32]. In our *in vitro* and *in vivo* studies, we found that treatment of cells with HF was associated with inactive CDK2 through up-regulation of p21^Cip1^, while ATS treatment inhibited CDK2 in association with the up-regulation of both p21^Cip1^ and p27^Kip1^. These data suggest that p21^Cip1^ and p27^Kip1^ are the key factors for the arrest of cancer cells at G1/G0 phase by the HF-ATS combination.

Next, we knocked down p21^Cip1^ or/and p27^Kip1^ in HCT116 cells or MCF-7 cells. In p21^Cip1^ knockdown cells, ATS or HF-ATS combination up-regulated p27^Kip1^ and inactivated CDK2, thereby resulting in cell arrest at G1/G0 phase. In contrast, HF treatment had no obvious effects on cell cycle progression in p21^Cip1^ knockdown cells. On the other hand, in p27^Kip1^ knockdown cells, HF or ATS arrested cells at G1/G0 phase, up-regulated p21^Cip1^, and inactivated CDK2. Notably, HF-ATS combination arrested more cells at the G1/G0 phase in p27^Kip1^ knockdown cells compared with either agent alone. Not surprisingly, p21^Cip1^ and p27^Kip1^ double knockdown completely abolished the growth inhibitory effects of HF, ATS, or HF-ATS combination. In conclusion, those results demonstrate that p21^Cip1^ and p27^Kip1^ are the two key factors for the ability of HF-ATS combination to arrest cancer cells at G1/G0 phase.

In summary, we reported the first study of the use of HF-ATS combination to treat cancer. Specifically, we investigated the effect of HF-ATS combination on cell cycle arrest at molecular, cellular and animal levels. Obviously, further work to develop HF-ATS combination into a useful clinical anticancer drug should continue, and the potential combinational drugs in TCM might provide the alternative “answers” for cancer treatment.

## MATERIALS AND METHODS

### Chemicals and antibodies

Halofuginone hydrobromide, RNase A, 5-Fluorouracil, puromycin, and Pierce (R) BCA Protein Assay Kit were obtained from Sigma-Aldrich (Munich, Germany). Artemisinin was purchased from Abcam (Cambridge, UK). Antibodies against p21^Waf1/Cip1^ (12D1), p27^Kip1^ (D69C12), phospho-CDK2 (Thr160), and β-Actin were purchased form Cell Signaling Technology (Danvers, MA). HRP-goat anti-rabbit secondary antibody was purchased from Invitrogen (Carlsbad, USA). Goat anti-mouse IgG-HRP secondary antibody was purchased from San Cruz Biotechnology (Santa Cruz, USA).

### Cell culture

HCT116, SW480, SW620, MCF-7, A375, MGC803, HepG2 and Hep3B cells were purchased from American Type Culture Collection (Manassas, USA). Cells were cultured in DMEM supplemented with 10% FBS in a humidified atmosphere containing 5% CO_2_ and 95% air at 37 °C. The medium was changed every three days, and cells were passaged using 0.05% trypsin/EDTA.

### MTT assay

The synergistic effect of HF-ATS combination on proliferation and viabilities of various cancer cell lines was determined by the 3-(4,5-dimethylthiazol-2-yl)-2,5-diphenyltetrazolium bromide (MTT) uptake method. Briefly, the cells (5,000 per well) were seeded in 96-well plates 24 h prior to treatment. The synergistic effect of HF and ATS on proliferation of different cell lines was determined in combinations of different concentrations of drugs. The optical density (O.D.) values for untreated group were set as 100% viability. Independent experiments were performed in triplicate.

### PI staining for cell cycle analysis

Cell cycle of cancer cells treated with HF, ATS, or both were analyzed. Briefly, HCT116 and MCF-7 cells were treated with HF, or ATS, or HF-ATS combination for 12 h and 24 h, respectively, then harvested and fixed in 80% ethanol and stored at −20 °C overnight. Cells were washed with PBS and stained with PI and RNase A (0.5 mg/mL) (in the dark) for 1 h. Data of cell cycle distribution was collected by using FACS Calibur flow cytometer and ModFit LT version 3.1 (Verity Software House, Topsham). In addition, cell synchronization by serum starvation was performed. After 24 h culture, HCT116 and MCF-7 cells were synchronized by serum starvation for 24 h and 48 h, respectively. The cells were then induced to re-enter the cell cycle by the addition of serum in the presence or absence of indicated drugs. After treatment with the compounds, the cells were analyzed as described above.

### Knockdown of p21^Cip1^ or/and p27^Kip1^

The PLKO.1 (puro) plasmid was purchased from Addgene (plasmid#8453), and the optimal 21mers for p21^Cip1^ and p27^Kip1^ were respectively selected from the RNAi consortium (TRC) (21mers for p21^Cip1^: 5′-CGCTCTACATCTTCTGCCTTA-3′; and 21mers for p27^Kip1^: 5′-GCGCAAGTGGAATTTCGATTT-3′). The 21mers were synthesized and inserted into PLKO.1 to generate the lentiviral plasmids, PLKO.1sh21 and PLKO.1sh27, according to the PLKO.1-TRC protocol [33]. Then HEK 293T cell was used for lentivirus package. Briefly, HEK-293T cells were plated in 6-well plates and changed to medium without penicillin-streptomycin before use. The lentiviral plasmids, together with the two viruses packaging plasmids (psPAX2 and pMD2.G, purchased from Addgene #12260 and #12259), were transfected into 293T cells by lipofectamine2000 (Invitrogen, 11668-019). The medium was changed on Day2, and the virus was collected on Days3 and 4. MCF-7 or HCT116 cells were infected with the virus in the presence of polybrene (8 μg/mL) for two days. Cells were then selected in fresh medium containing puromycin (3 μg/mL) for 3–5 days. The puromycin-resistant cells were pooled and the knockdown efficiency was verified by both quantitative real-time RT-PCR and/or western blot analyses. For of p21^Cip1^ and p27^Kip1^ double knockdown, the p27^Kip1^ knockdown cell lines were used to further knockdown p21^Cip1^.

### Western blot analysis

HCT116 cells or MCF-7 cells were treated with drugs for 12 h or 24 h, respectively, and whole cell lysates were then obtained by suspending the cells in lysis buffer. Followed by centrifugation at 13,500 rpm for 15 min at 4 °C, total protein concentration was measured using Pierce (R) BCA Protein Assay Kit, and 10 to 25 μg of protein was separated on 10% SDS-PAGE and transferred to PVDF membranes. After blocking (5% skim milk powder in TBST, 20) for 1 h at room temperature, the membrane was then incubated with primary antibody overnight at 4 °C. Afterwards, the membrane was incubated with secondary antibody for 1 h at room temperature. All antibodies were diluted in TBS-Tween 20 containing 5% dry milk. The immune-reactive proteins were detected by enhanced chemiluminescence (ECL) using X-ray film and ECL reagent.

### Quantitative analysis of mRNA level

Total RNA was isolated from HCT116 cells or MCF-7 cells using TRIzol reagent (Invitrogen), and cDNAs were subsequently prepared by reverse transcription. Quantitative polymerase chain reaction (PCR) was performed using the Quantitect SYBR green PCR Master mix (Qiagen, Valencia, CA) with 1 μL cDNA in a final volume of 10 μL and the following primers at a final concentration of 1000 nM. Primers for p21^Cip1^ were 5′-GCAGACCAGCATGACAGATTT-3′ (forward) and 5′-GATGTAGAGCGGGCCTTTGA-3′ (reverse). Primers for p27^Kip1^ were 5′- GGCAAGTACGAGTGGCAAGA-3′ (forward) and 5′-AGAAGAATCGTCGGTTGCAGG-3′ (reverse).

Amplification of p21^Cip1^ and p27^Kip1^ cDNAs was performed using the LightCycler 2000 instrument (Roche, Indianapolis, IN). The cycling conditions comprised a denaturation step for 15 minutes at 95 °C, followed by 40 cycles of denaturation (95 °C for 15 seconds), annealing (59 °C for 20 seconds), and extension (72 °C for 15 seconds). After amplification, a melting curve analysis was performed with denaturation at 95 °C for 5 seconds, then continuous fluorescence measurement was made from 70 °C to 95 °C at 0.1 °C/second. Each sample was amplified in duplicate.

### Tumorigenesis in nude mice

Six-week old female BALB/c nude mice were purchased from the Laboratory Animal Services Centre, The Chinese University of Hong Kong. Mice were kept at room temperature 23°C ± 2°C with an alternating 12 h light-dark cycle, and were allowed access to food and water. All of the experimental protocols were carried out with the approval of the Committee on Use of Human and Animal Subjects in Teaching and Research of Hong Kong Baptist University and according to the Regulations of the Department of Health, Hong Kong SAR, China. HCT116 cells (5 × 10^6^ cells per mouse) were suspended in PBS and inoculated subcutaneously into the left flank of each mouse; tumor growth was monitored regularly. Once tumors were palpable, (~100 mm^3^), mice were divided at random into five groups, each group with 5 mice, treated as follows: (1) vehicle group (daily i.p. saline), (2) HF group (daily i.p. 5 μg/kg of HF), (3) ATS group (daily i.p. 50 mg/kg of ATS), (4) HF combined with ATS group (daily i.p. 5 μg/kg of HF and 50 mg/kg of ATS), (5) 5Fu group (daily i.p. 10 mg/kg of 5Fu). Although much higher dosage of HF and ATS were given to mouse *in vivo* compared with cells *in vitro*, the concentration ratio of HF-ATS combination was equal. The tumors were measured with calipers every day, and tumor volumes were calculated by the following formula: a^2^×b×0.4, where “a” is the smallest diameter and “b” is the diameter perpendicular to “a”. At the end of the experiment, the mice were sacrificed and the tumor weights of each animal were analyzed.

### H&E staining and protein extraction form tumors

After the mice were sacrificed, tumors were resected immediately and fixed in 10% neutral buffered paraformaldehyde at 4 °C for 24 h. Selected samples were embedded in paraffin, sectioned and treated in the following steps for H&E staining: hematoxylin for 10 min, 1% acid–ethanol for 30 s, 1% ammonia water for 30 s, and eosin for 10 s. After staining, tissue sections were dehydrated with water–ethanol–xylene gradients. Sections were finally mounted with D.P.X mountant(Sigma, 317616) for histology analysis. To measure the p21^Cip1^, p27^Kip1^ and phospho-CDK2 activity in tumor tissue, tumors from every group were collected and dispersed in lysis buffer by sonication for protein extraction. After centrifugation at 13,500 rpm for 15 min at 4 °C, the supernatant was collected and regarded as the total soluble proteins which were subsequently subjected to western blot analysis.

### Immunofluorescence

Selected samples which had been embedded in paraffin were sectioned and stained with p21^Waf1/Cip1^ (12D1), p27^Kip1^ (D69C12), and phospho-CDK2 (Thr160). All primary antibodies were used at 1:100. After incubation overnight 4 °C for 24 h, the sections were then incubated with flurochrome-conjugated secondary antibody for 1 h and cover-slipped slides with DAPI for 10 min. The sections were finally mounted with D.P.X mountant (Sigma, 317616) for analysis.

### Statistical analysis

Each experiment was performed at least three times. GraphPad Prism 5.0 software was used for statistic analysis. Combination Index was calculated by CompuSyn software 2.0 according to the software's instruction.

## SUPPLEMENTARY MATERIALS FIGURES



## References

[1] Knox SS (2010). From ‘omics’ to complex disease: a systems biology approach to gene-environment interactions in cancer. Cancer Cell Int.

[2] Huang L, Li F, Sheng J, Xia X, Ma J, Zhan M, Wong ST (2014). DrugComboRanker: drug combination discovery based on target network analysis. Bioinformatics.

[3] Azmi AS, Wang Z, Philip PA, Mohammad RM, Sarkar FH (2010). Proof of concept: network and systems biology approaches aid in the discovery of potent anticancer drug combinations. Mol Cancer Ther.

[4] Sun Y, Sheng Z, Ma C, Tang K, Zhu R, Wu Z, Shen R, Feng J, Wu D, Huang D, Huang D, Fei J, Liu Q, Cao Z (2015). Combining genomic and network characteristics for extended capability in predicting synergistic drugs for cancer. Nat Commun.

[5] Wang L, Zhou GB, Liu P, Song JH, Liang Y, Yan XJ, Xu F, Wang BS, Mao JH, Shen ZX, Chen SJ, Chen Z (2008). Dissection of mechanisms of Chinese medicinal formula Realgar-Indigo naturalis as an effective treatment for promyelocytic leukemia. Proc Natl Acad Sci U S A.

[6] Yang HJ, Shen D, Xu HY, Lu P (2012). A new strategy in drug design of Chinese medicine: theory, method and techniques. Chin J Integr Med.

[7] Tin AS, Sundar SN, Tran KQ, Park AH, Poindexter KM, Firestone GL (2012). Antiproliferative effects of artemisinin on human breast cancer cells requires the downregulated expression of the E2F1 transcription factor and loss of E2F1-target cell cycle genes. Anti-cancer drugs.

[8] Zhu S, Liu W, Ke X, Li J, Hu R, Cui H, Song G (2014). Artemisinin reduces cell proliferation and induces apoptosis in neuroblastoma. Oncology reports.

[9] Tran KQ, Tin AS, Firestone GL (2014). Artemisinin triggers a G1 cell cycle arrest of human Ishikawa endometrial cancer cells and inhibits cyclin-dependent kinase-4 promoter activity and expression by disrupting nuclear factor-kappaB transcriptional signaling. Anti-cancer drugs.

[10] Chen GQ, Tang CF, Shi XK, Lin CY, Fatima S, Pan XH, Yang DJ, Zhang G, Lu AP, Lin SH, Bian ZX (2015). Halofuginone inhibits colorectal cancer growth through suppression of Akt/mTORC1 signaling and glucose metabolism. Oncotarget.

[11] Leiba M, Jakubikova J, Klippel S, Mitsiades CS, Hideshima T, Tai YT, Leiba A, Pines M, Richardson PG, Nagler A, Anderson KC (2012). Halofuginone inhibits multiple myeloma growth in vitro and in vivo and enhances cytotoxicity of conventional and novel agents. British journal of haematology.

[12] Gnainsky Y, Spira G, Paizi M, Bruck R, Nagler A, Genina O, Taub R, Halevy O, Pines M (2006). Involvement of the tyrosine phosphatase early gene of liver regeneration (PRL-1) in cell cycle and in liver regeneration and fibrosis effect of halofuginone. Cell and tissue research.

[13] Chou TC (2010). Drug combination studies and their synergy quantification using the Chou-Talalay method. Cancer research.

[14] Evan GI, Vousden KH (2001). Proliferation, cell cycle and apoptosis in cancer. Nature.

[15] Hartwell LH, Kastan MB (1994). Cell cycle control and cancer. Science.

[16] Kohn KW, Jackman J, O'Connor PM (1994). Cell cycle control and cancer chemotherapy. Journal of cellular biochemistry.

[17] Hill BT, Baserga R (1975). The cell cycle and its significance for cancer treatment. Cancer treatment reviews.

[18] Vilhelmova N, Jacquet R, Quideau S, Stoyanova A, Galabov AS (2011). Three-dimensional analysis of combination effect of ellagitannins and acyclovir on herpes simplex virus types 1 and 2. Antiviral research.

[19] Chou TC (2006). Theoretical basis, experimental design, and computerized simulation of synergism and antagonism in drug combination studies. Pharmacological reviews.

[20] Toyoshima H, Hunter T (1994). p27, a novel inhibitor of G1 cyclin-Cdk protein kinase activity, is related to p21. Cell.

[21] Komarova NL, Boland CR (2013). Cancer: calculated treatment. Nature.

[22] Keith CT, Borisy AA, Stockwell BR (2005). Multicomponent therapeutics for networked systems. Nat Rev Drug Discov.

[23] Qiu J (2007). ‘Back to the future’ for Chinese herbal medicines. Nat Rev Drug Discov.

[24] Juarez P, Mohammad KS, Yin JJ, Fournier PG, McKenna RC, Davis HW, Peng XH, Niewolna M, Javelaud D, Chirgwin JM, Mauviel A, Guise TA (2012). Halofuginone inhibits the establishment and progression of melanoma bone metastases. Cancer research.

[25] Gu Y, Wu G, Wang X, Wang X, Wang Y, Huang C (2014). Artemisinin prevents electric remodeling following myocardial infarction possibly by upregulating the expression of connexin 43. Mol Med Rep.

[26] Nagler A, Katz A, Aingorn H, Miao HQ, Condiotti R, Genina O, Pines M, Vlodavsky I (1997). Inhibition of glomerular mesangial cell proliferation and extracellular matrix deposition by halofuginone. Kidney Int.

[27] Cao Q, Jiang Y, Shi J, Liu X, Chen J, Niu T, Li X (2015). Artemisinin inhibits tumour necrosis factor-alpha-induced vascular smooth muscle cell proliferation in vitro and attenuates balloon injury-induced neointima formation in rats. Clin Exp Pharmacol Physiol.

[28] Morgan DO (1995). Principles of CDK regulation. Nature.

[29] van den Heuvel S, Harlow E (1993). Distinct roles for cyclin-dependent kinases in cell cycle control. Science.

[30] Wadler S (2001). Perspectives for cancer therapies with cdk2 inhibitors. Drug resistance updates: reviews and commentaries in antimicrobial and anticancer chemotherapy.

[31] Orlando S, Gallastegui E, Besson A, Abril G, Aligue R, Pujol MJ, Bachs O (2015). p27Kip1 and p21Cip1 collaborate in the regulation of transcription by recruiting cyclin-Cdk complexes on the promoters of target genes. Nucleic acids research.

[32] Martin A, Odajima J, Hunt SL, Dubus P, Ortega S, Malumbres M, Barbacid M (2005). Cdk2 is dispensable for cell cycle inhibition and tumor suppression mediated by p27(Kip1) and p21(Cip1). Cancer cell.

[33] Stewart SA, Dykxhoorn DM, Palliser D, Mizuno H, Yu EY, An DS, Sabatini DM, Chen IS, Hahn WC, Sharp PA, Weinberg RA, Novina CD (2003). Lentivirus-delivered stable gene silencing by RNAi in primary cells. RNA.

