# Supplementary material for: Halofuginone and artemisinin synergistically arrest cancer cells at the G1/G0 phase by upregulating p21Cip1 and p27Kip1
Source: Oncotarget. 2016 Jul 1;7(31):50302–14. doi: 10.18632/oncotarget.10367 (PMC5226584; doi:10.18632/oncotarget.10367)
Supplement: Supplementary file 1 [file oncotarget-07-50302-s001.pdf]

Halofuginone and artemisinin synergistically arrest cancer cells at the G1/G0 phase by upregulating p21^{Cip1} and p27^{Kip1}

SUPPLEMENTARY FIGURES

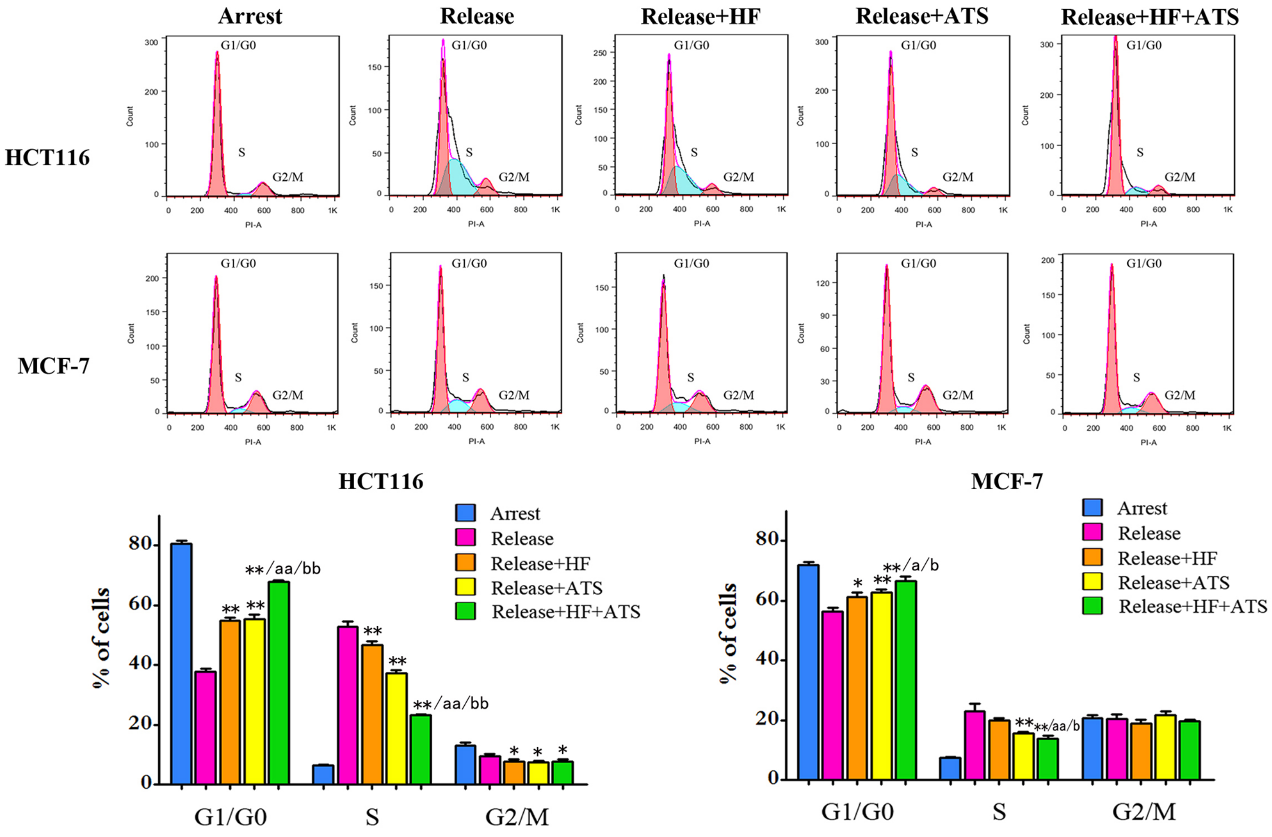

Supplementary Figure 1: HF-ATS combination markedly inhibited cell progression from G1 to S phase in serum starvation-release experiment. Flow cytometry (upper panel) and histogram (bottom panel) analysis of HCT116 and MCF-7 cells treated with the combination of HF (10 nM) and ATS (160 μM) for 12 h and 24 h respectively. *$P < 0.05$, **$P < 0.01$, compared with control group; a $P < 0.05$, aa $P < 0.01$, compared with HF; b $P < 0.05$, bb $P < 0.01$, compared with ATS.

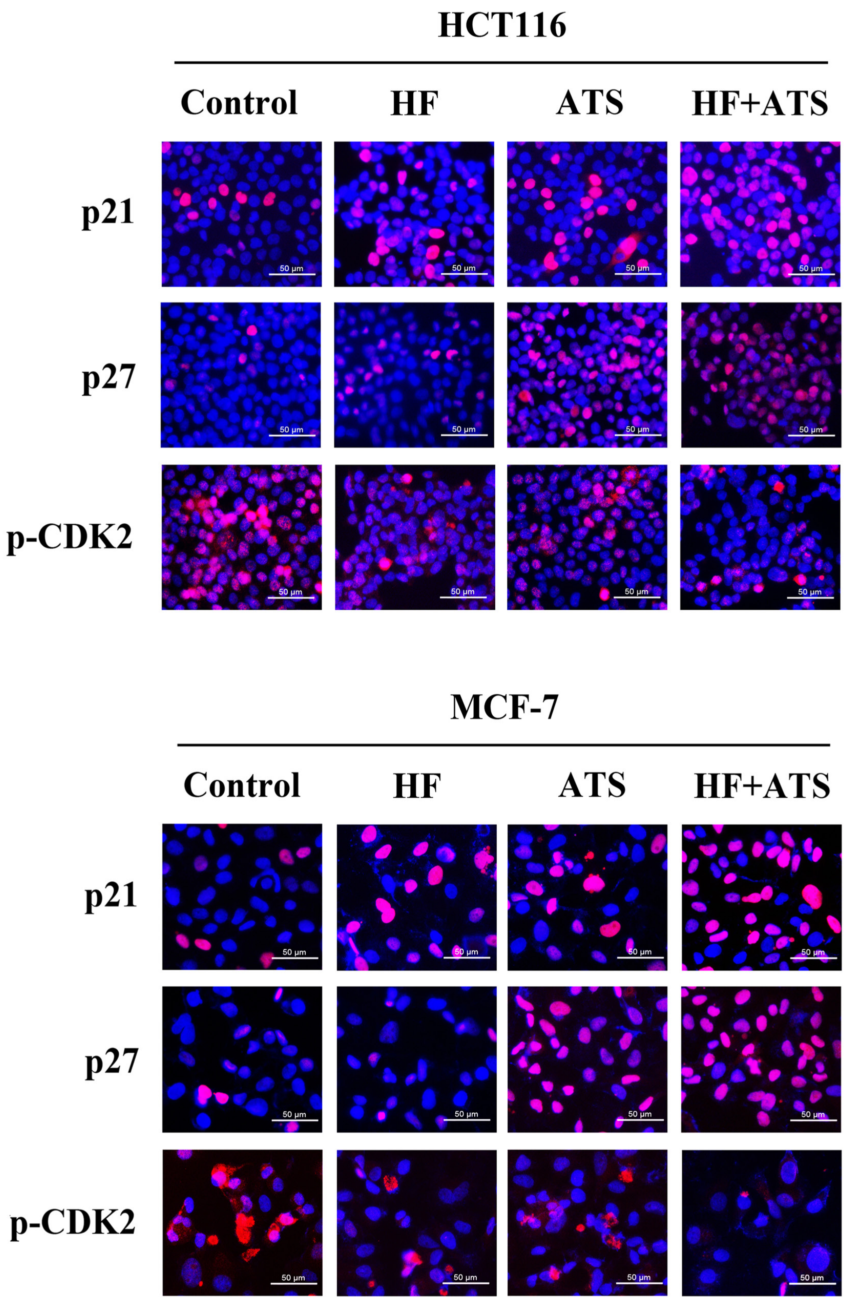

Supplementary Figure 2: HF-ATS combination markedly induced nuclear accumulation of p21^{Cip1} and p27^{Kip1} in HCT116 cells and MCF-7 cells. Immunofluorescence staining of p21^{Cip1}, p27^{Kip1} and phospho-CDK2 in HCT116 cells and MCF-7 cells treated with HF, ATS, or HF-ATS combination. Blue: DAPI, Red: p21^{Cip1}, p27^{Kip1} or p-CDK2. Scale bar = 50 µm.

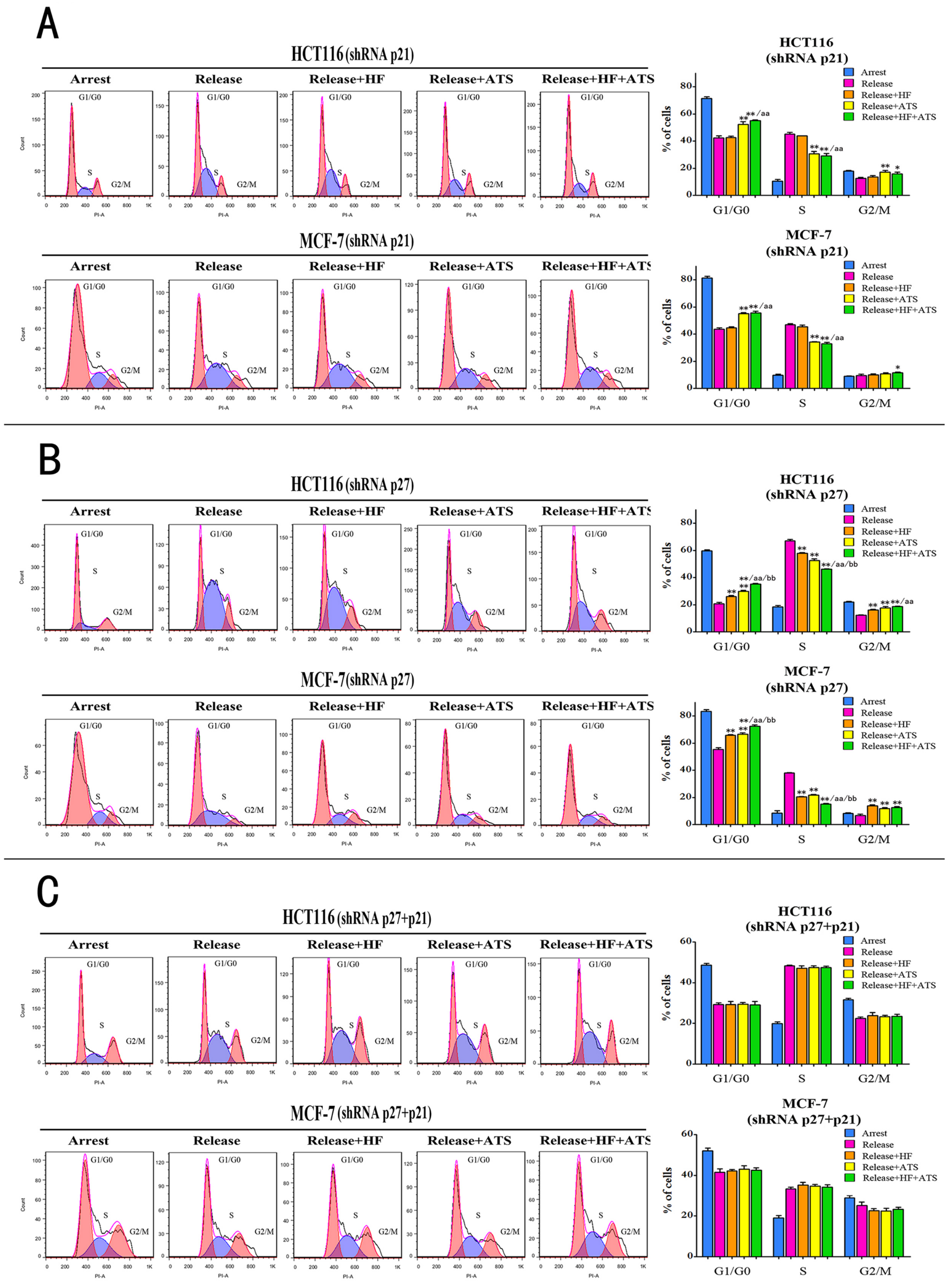

Supplementary Figure 3: Cell cycle progressions were markedly inhibited by HF-ATS combination in p21^{Cip1} or/and p27^{Kip1} knockdown cells under starvation-release. A. Flow cytometry (left panel) and histogram (right panel) analysis of p21^{Cip1} knockdown HCT116 cells and MCF-7 cells treated with the combination of HF (10 nM) and ATS (160 µM) for 12 h or 24 h, respectively. **B.** Flow cytometry (left panel) and histogram (right panel) analysis of p27^{Kip1} knockdown HCT116 cells and MCF-7 cells treated with the combination of HF (10 nM) and ATS (160 µM) for 12 h or 24 h, respectively. **C.** Flow cytometry (left panel) and histogram (right panel) analysis of p21^{Cip1}-p27^{Kip1} double knockdown HCT116 cells and MCF-7 cell lines treated with combination of HF (10 nM) and ATS (160 µM) for 12 h or 24 h, respectively. *P < 0.05, **P < 0.01, compared with control group; a P < 0.05, aa P < 0.01, compared with HF; b P < 0.05, bb P <0.01, compared with ATS.

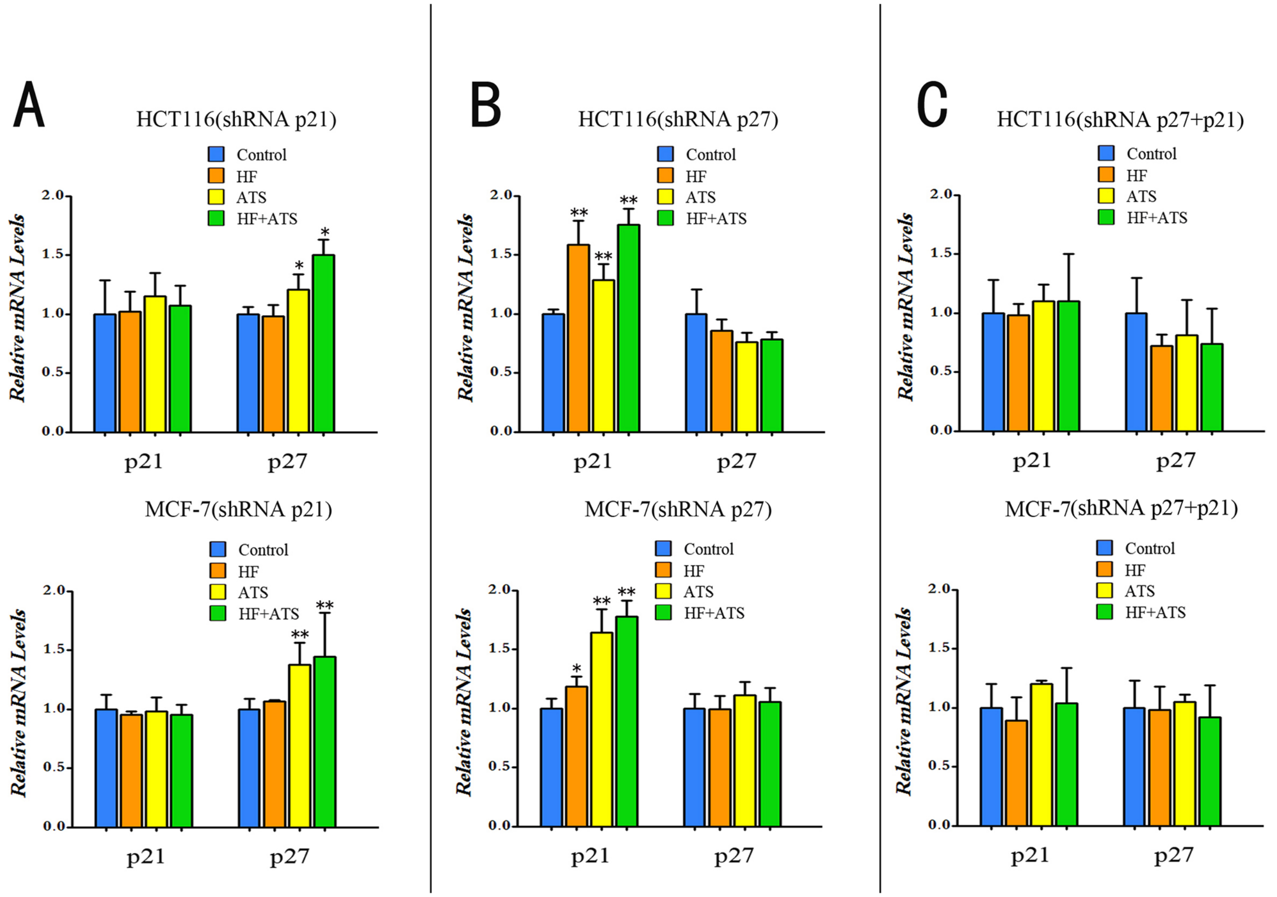

Supplementary Figure 4: Effects of HF-ATS combination on mRNA of $p21^{Cip1}$ and $p27^{Kip1}$ in knockdown cancer cells. **A.** The mRNA of $p27^{Kip1}$ were up-regulated by combination of HF (10 nM) and ATS (160 μM) in $p21^{Cip1}$ knockdown HCT116 cells and MCF-7 cells. **B.** The mRNA of $p21^{Cip1}$ were up-regulated by combination of HF (10 nM) and ATS (160 μM) in $p27^{Kip1}$ knockdown HCT116 cells and MCF-7 cells. **C.** Combination of HF (10 nM) and ATS (160 μM) had no effects on mRNA of $p21^{Cip1}$ and $p27^{Kip1}$ in $p21^{Cip1}$-$p27^{Kip1}$ double knockdown HCT116 cells and MCF-7 cell lines.

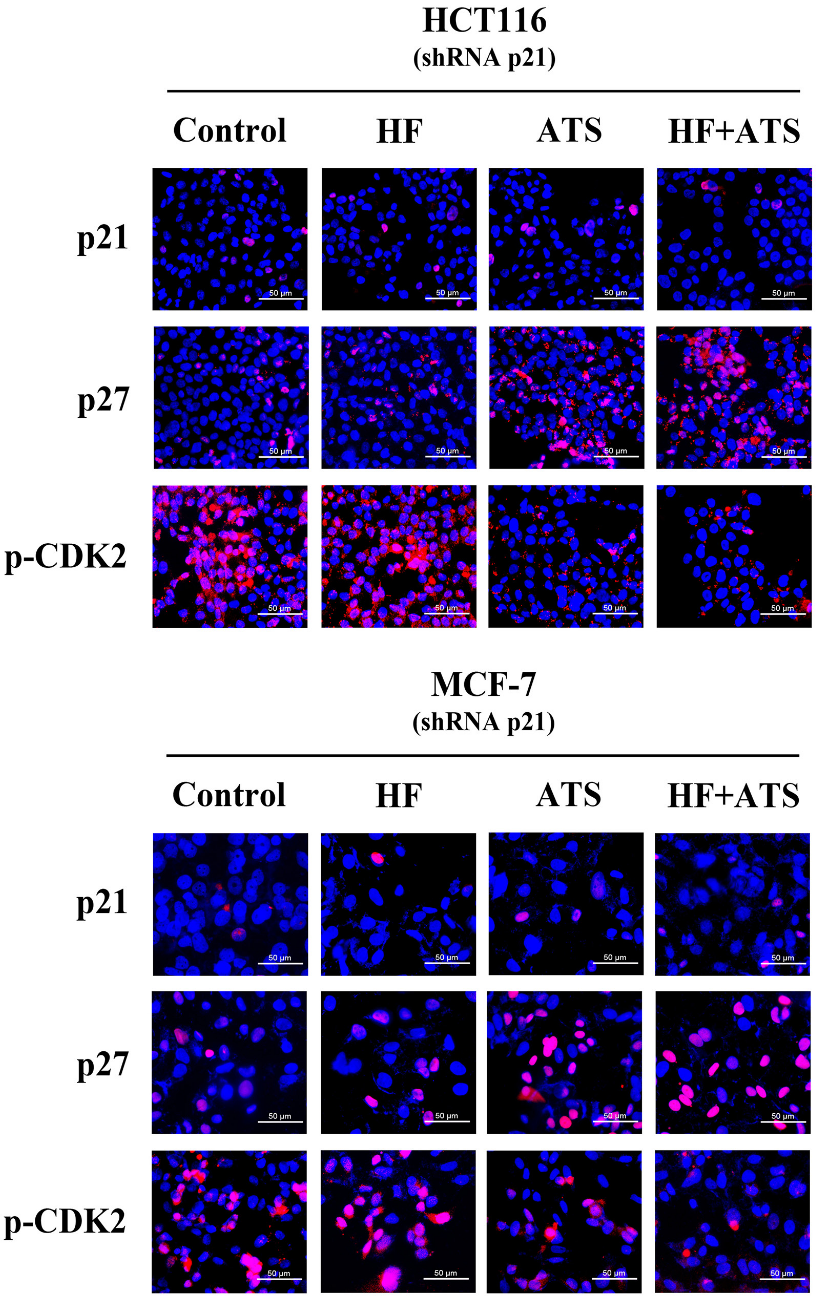

Supplementary Figure 5: HF-ATS combination markedly induced nuclear accumulation of $p27^{Kip1}$ in $p21^{Cip1}$ knockdown HCT116 cells and MCF-7 cells. Immunofluorescence staining of $p21^{Cip1}$, $p27^{Kip1}$ and phospho-CDK2 in $p21^{Cip1}$ knockdown HCT116 cells and MCF-7 cells. Scale bar = 50 µm.

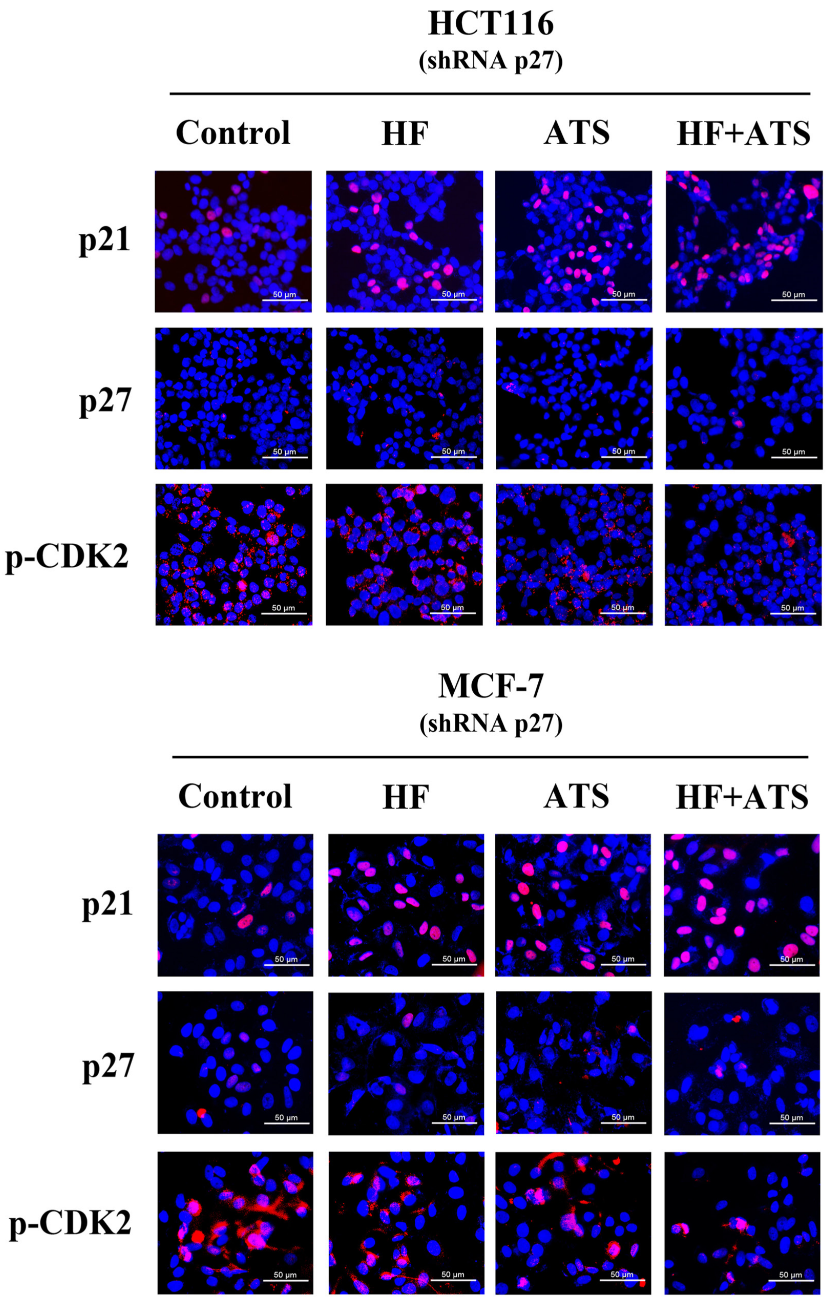

Supplementary Figure 6: HF-ATS combination markedly induced nuclear accumulation of p21^{Cip1} in p27^{Kip1} knockdown HCT116 cells and MCF-7 cells. Immunofluorescence staining of p21^{Cip1}, p27^{Kip1} and phospho-CDK2 in p27^{Kip1} knockdown HCT116 cells and MCF-7 cells. Scale bar = 50 μm.

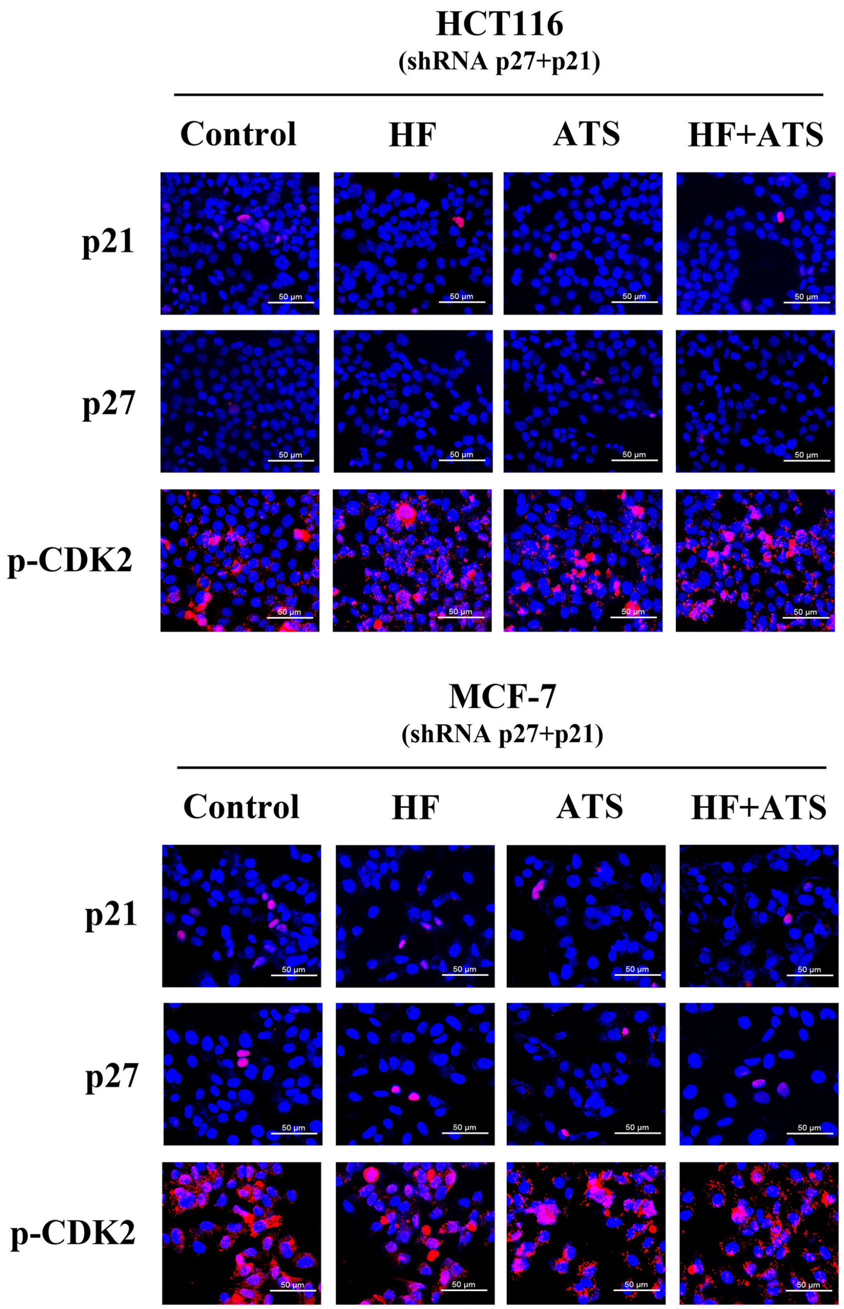

Supplementary Figure 7: HF-ATS combination failed to induce nuclear accumulation of $p21^{Cip1}$ and $p27^{Kip1}$ in $p21^{Cip1}$-$p27^{Kip1}$ double knockdown HCT116 cells and MCF-7 cells. Immunofluorescence staining of $p21^{Cip1}$, $p27^{Kip1}$ and phospho-CDK2 in $p21^{Cip1}$-$p27^{Kip1}$ double knockdown HCT116 cells and MCF-7 cells. Scale bar = 50 µm.

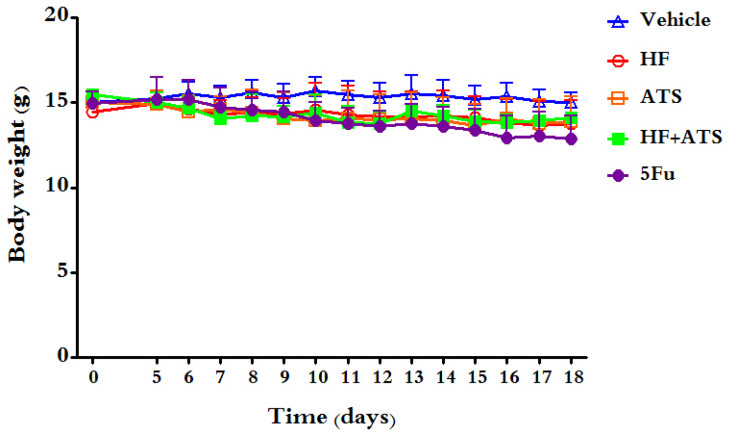

Supplementary Figure 8: Bodyweight changes in 5 groups of xenograft nude mice treated with vehicle, HF, ATS, HF-ATS, and 5Fu.
